# Efficacy of Compound Kushen Injection in Combination with Induction Chemotherapy for Treating Adult Patients Newly Diagnosed with Acute Leukemia

**DOI:** 10.1155/2016/3121402

**Published:** 2016-09-21

**Authors:** Honglei Tu, Bo Lei, Shan Meng, Hailing Liu, Yongchang Wei, Aili He, Wanggang Zhang, Fuling Zhou

**Affiliations:** ^1^Department of Clinical Hematology, Zhongnan Hospital, Wuhan University, Wuhan, Hubei Province 430071, China; ^2^Department of Clinical Hematology, Second Affiliated Hospital, Medical School of Xi'an Jiaotong University, Xi'an, Shaanxi Province 710004, China; ^3^Department of Radiation and Medical Oncology, Zhongnan Hospital, Wuhan University, Wuhan, Hubei Province 430071, China

## Abstract

We assessed the clinical effectiveness and safety of CKI (compound Kushen injection) plus standard induction chemotherapy for treating adult acute leukemia (AL). We randomly assigned 332 patients with newly diagnosed AL to control (*n* = 165, receiving DA (daunorubicin and cytarabine) or hyper-CVAD (fractionated cyclophosphamide, doxorubicin, vincristine, and dexamethasone)) or treatment (*n* = 167, receiving CKI and DA or hyper-CVAD) groups. Posttreatment, treatment group CD3+, CD4+, CD4+/CD8+, natural killer (NK) cell, and immunoglobulin (IgG, IgA, and IgM) levels were significantly higher than those of the control group (*p* < 0.05), and CD8+ levels were lower in the treatment group than in the control group (*p* < 0.05). Treatment group interleukin- (IL-) 4 and IL-10 levels were significantly higher compared to the control posttreatment (both* p* < 0.05) as were complete remission, overall response, and quality of life (QoL) improvement rates (*p* < 0.05). The control group had more incidences of grade 3/4 hematologic and nonhematologic toxicity (*p* < 0.05). Responses to induction chemotherapy, QoL improvement, and adverse events incidence between control group patients with acute myeloid leukemia and acute lymphocytic leukemia were not significantly different. CKI plus standard induction chemotherapy is effective and safe for treating AL, possibly by increasing immunologic function.

## 1. Introduction

Acute leukemia (AL), a malignant neoplasm, is characterized by clonal blood cell proliferation within the bone marrow. Historically, AL diagnosis is linked with poor prognosis, particularly in older adults. Improved disease treatment and management have led to increased overall survival trends [1]. The Surveillance Epidemiology and End Results reported 24% and 65% 2002–2008 relative five-year survival rates in adults with acute myeloid leukemia (AML) or acute lymphoblastic leukemia (ALL), respectively [2]. The nature of adult AL necessitates emergent, aggressive in-patient chemotherapy. However, standard chemotherapy is not always tolerated by patients; the adverse events during chemotherapy, such as myelosuppression, gastrointestinal reaction, infection, and cardiotoxicity, often lead to interruption of chemotherapy [3]; consequently, there is treatment failure. Therefore, such patients urgently need effective, low-toxicity therapy.

Therapy integrating traditional Chinese and Western medicine has been the most distinctive method for treating malignant tumors in China [4–7]. As a representative of traditional Chinese medicine (TCM), compound Kushen injection (CKI) is extracted from the Kushen (*Radix Sophorae Flavescentis*) and Baituling (*Rhizoma smilacis Glabrae*) herbs; its primary components are oxymatrine and matrine [8, 9]. Additional File 1 (in Supplementary Material available online at http://dx.doi.org/10.1155/2016/3121402) contains the CKI fingerprint. The State Food and Drug Administration of China approved CKI for treating cancer in 1992 [10]. Since then, CKI has been extensively used in the Chinese clinical setting. There are many clinical reports demonstrating its anticancer effect and these reports include using CKI to treat gastric cancer, liver cancer, lung cancer, breast cancer, ovarian cancer, colorectal cancer, and other cancer types [11–14]. It is reported that CKI attenuates chemotherapy and radiotherapy side effects by improving quality of life (QoL), regulating immune function, and synergizing chemotherapy and radiotherapy therapeutic effects [15, 16]. In addition, it is reported that there is a positive effect of CKI on bone cancer pain: compared with radiotherapy or bisphosphonates, CKI showed significant effects on the improvement of pain relief in patients with bone cancer pain and the increase in Karnofsky Performance Status (KPS). The patients treated with CKI achieved statistically significant reductions in the incidence of leukopenia and nausea. No severe adverse events were found and no treatment was stopped because of adverse events of CKI in the treatment groups [17]. However, the scientific literature contains scarce empirical data involving CKI in ALs, and the underlying complex mechanisms of its anticancer effect are not fully understood. In the study, our primary aim was to determine whether CKI with induction chemotherapy has a similar or better effect in improving the objective response rate (ORR) in adult AL. The secondary aims were to compare immunologic function, quality of life (QoL), clinical symptoms, and adverse drug reactions (ADRs) and to explore the reasons therein.

## 2. Materials and Methods

### 2.1. Study Groups

We selected 332 previously untreated patients (aged 18–78 years) with AL who were treated at our hospital between January 2011 and January 2016. According to the World Health Organization criteria, 175 and 157 patients were diagnosed with AML and ALL, respectively. The patients were randomly assigned to a treatment (*n* = 167) or control (*n* = 165) group. No patient had central nervous system diseases, other malignancies, or uncontrolled infections. They also had no inflammatory conditions, pathological antecedents, or endocrine dysfunctions. We excluded patients with known confusion or who were deemed too ill to participate. The Wuhan University Ethics Committee approved this study and it met international standards for patient confidentiality. All patients signed informed consent forms according to the Declaration of Helsinki.

### 2.2. Treatment Methods

Both groups received induction chemotherapy, in which patients with confirmed AML received the DA regimen, and patients with ALL received the hyper-CVAD regimen. The treatment group received CKI in addition to the DA or hyper-CVAD regimens.

The DA regimen consisted of 60 mg/m^2^ daunorubicin on days 1–3 and 200 mg/m^2^ cytarabine on days 1–7. Hyper-CVAD treatment consisted of 300 mg/m^2^ fractionated cyclophosphamide twice daily on days 1–3, 50 g/m^2^ doxorubicin on day 4, 2 mg vincristine on days 4 and 11, and 40 mg dexamethasone on days 1–4 and 11–14. In the treatment group, according to the instructions, 20 mL CKI (Shanxi Zhendong Pharmacy Limited Company, Chinese medicine permit number Z14021231) plus 200 mL saline was administered by intravenous drip, at 40–60 drips each min, once daily for 14 days. After the end of the first induction course, a bone marrow biopsy was used to assess the treatment response. Patients with complete remission (CR) received continued consolidation therapy or autologous or allogeneic stem cell transplantation, and patients who did not achieve CR in 2 cycles had their induction programs adjusted.

### 2.3. Observation Indices

The present analysis involved only 1-2 cycle of induction chemotherapy. Treatment response, QoL, and toxicity (hematologic and nonhematologic (including gastrointestinal reaction, pneumonia, hepatotoxicity, neurological dysfunction, renal dysfunction, and skin reactions)) were evaluated. One day before treatment and 1 week after the end of the treatment course in both groups, the peripheral blood T-lymphocyte subgroup (CD3+, CD4+, and CD8+), natural killer (NK) cell, and immunoglobulin (IgG, IgA, and IgM) levels were detected with flow cytometry, the CD4+/CD8+ ratios were calculated, and cytokines (interleukin- (IL-) 4 and IL-10) were detected by enzyme-linked immunosorbent assay (ELISA).

### 2.4. Statistical Analysis

We used SPSS version 20.0 for the statistical analyses. All data are presented as the mean ± standard deviation (*x* ± *s*). The groups were compared using independent sample* t*-tests. The differences in characteristics between the two groups were examined using the *χ* ^2^ test.* p* < 0.05 was considered significant.

## 3. Results

### 3.1. Baseline Characteristics

The clinicopathological information of the treatment and control groups did not significantly differ (*p* > 0.05, Table 1).

### 3.2. Changes in Immune Function Indices

The peripheral blood CD3+, CD4+, CD8+, CD4+/CD8+, NK cell, and immunoglobulin levels in the treatment and control groups did not differ significantly 1 day before treatment (*p* > 0.05). After the first course of induction chemotherapy, in the control group, CD3+, CD4+, CD4+/CD8+, NK cell, and immunoglobulin (IgA, IgM, and IgG) levels decreased significantly (*p* < 0.05) and CD8+ was increased significantly (*p* < 0.05); in the treatment group, levels of CD3+, IgA, and IgM decreased significantly (*p *< 0.05), CD4+ was increased significantly (*p *< 0.05), and CD8+, CD4+/CD8+, NK cell, and IgG levels did not change significantly (*p* > 0.05). This suggests that immunity function has been decreased in AL patients with induction chemotherapy. The serum CD3+, CD4+, CD4+/CD8+, NK cells, and immunoglobulin levels were significantly lower in the control group than in the treatment group (*p* < 0.05, Table 2), which implies that CKI improves immunologic function in patients with AL.

### 3.3. Cytokine Changes

Before treatment, the treatment and control groups did not significantly differ in the peripheral blood counts for IL-4 and IL-10 (*p* > 0.05). After the end of treatment, IL-4 and IL-10 levels were increased significantly in the treatment group and were significantly higher than those of the control group (both* p* < 0.05); however, those of the control group were decreased significantly compared to pretreatment levels (*p* < 0.05, Table 2).

### 3.4. Response to Induction Chemotherapy

Our evaluation was based on the National Comprehensive Cancer Network (NCCN) Guidelines (Version 1. 2014) for evaluating response criteria for blood and bone marrow in AL: CR (no circulating blasts or extramedullary disease, trilineage hematopoiesis (TLH) < 5% blasts, absolute neutrophil count (ANC) > 1000/*μ*L, platelets > 1000,000/*μ*L, and no recurrence for 4 weeks); CRi (CR with incomplete blood count recovery: recovery of platelets but <1000,000 or ANC = 1000/*μ*L); ORR (ORR = CR + CRi). We assessed disease status via bone marrow biopsies performed 14 days (±2 days) after induction chemotherapy. After the first cycle of induction chemotherapy, the treatment group ORR rate was significantly higher than that of the control (AML: 92% versus 73.6%,* p* < 0.05; ALL: 89.9% versus 69.2%,* p* < 0.05) (Table 3), which showed that CKI plus chemotherapy significantly improved the ORR as compared with induction chemotherapy alone.

### 3.5. Evaluation of QoL

QoL evaluation was according to the Karnofsky Performance Status (KPS) and was classified as improvement (improved KPS of ≥10 points posttreatment); stabilization (improved or decreased KPS of <10 points posttreatment); deterioration (decreased KPS of ≥10 points posttreatment). Before treatment, the two groups patients did not significantly differ in KPS (*p* > 0.05, Table 1). After completing the first cycle of induction therapy, the treatment group KPS improvement rate was significantly higher than that of the control (AML: 55.7% versus 28.7%,* p* < 0.05; ALL: 59.5% versus 24.4%,* p* < 0.05), and KPS deterioration rate was significantly lower in the treatment group than in the control (AML: 9.1% versus 28.7%,* p* < 0.05; ALL: 11.4% versus 38.5%,* p* < 0.05) (Table 4), indicating that CKI plus chemotherapy can improve patient QoL.

### 3.6. Toxicity Reactions

According to the National Cancer Institute (NCI) Common Terminology Criteria for Adverse Events (CTCAE) v 3.0 [18], drug toxicity and adverse reactions were classified into four grades (1–4). Grade 1/2 adverse reactions were observed in many patients and were not considered in this study.

Whether in patients with AML or ALL, there was more hematologic toxicity in the control group (*p* < 0.05). Thrombocytopenia and anemia were observed in 9 patients (5.4%) in the treatment group as compared with 38 patients (23%) in the control group, and the patients received blood platelet or red blood cell transfusions (*p* < 0.05). Similarly, nonhematologic toxicity occurred significantly more often in the control group. In total, 2 patients (1.2%) in the treatment group compared with 15 patients (9.1%) in the control group developed grades 3 and 4 pneumonia (*p* < 0.05); 5 control group patients died of pneumonia during treatment; no treatment group patient died. The incidences of adverse events between patients with AML and ALL in the same treatment groups were not significantly different (Table 5).

The results show that chemotherapy-related complications are reduced following CKI plus chemotherapy as compared with chemotherapy alone.

## 4. Discussion

Traditional Chinese Medicine (TCM) holds that, in treating tumors, eliminating pathogenic factors and strengthening genuine Qi are of equal importance: eliminating pathogenic factors is killing tumor cells by using radiotherapy or chemotherapy and strengthening genuine Qi protects immunologic functions of the organism by using drugs, increasing immunity of the organism [19, 20]. Hematopoietic stem cells that have lost the normal differentiation capacity into mature blood cells give rise to leukemia [21, 22]. In AL treatment, induction chemotherapy to achieve CR is considered basic therapy [23]. However, chemotherapy cannot avoid injuring the immune system, which mainly manifests in the fact that chemotherapy drugs directly or indirectly kill the immunologic effector cells, leading to decreased immune function [24, 25]. Antitumor immune responses are T-cell-mediated specific responses. T-cells can be divided into CD4+ helper/induced T-cells and CD8+ suppressor/killer T-cells at a CD4+/CD8+ ratio of 1.5–2.0. When the immunologic function in patients with cancer is impaired, CD4+ cells will decrease, CD8+ cells will increase, and the CD4+/CD8+ ratio decreases or even inverts [26]. CD3+ cells reflect the total CD4 and CD8 levels. When cellular immunologic function decreases, the NK cells ratio will also decrease, as they are unable to effectively kill tumor cells. Besides, immunoglobulins (Ig) also play an important role in adaptive immunity [27]. Therefore, flow cytometry determination of the changes in peripheral blood T-cell subgroups, NK cells, and immunoglobulins (IgG, IgA, and IgM) can illustrate the changes in immunologic function.

In our study, T-cell subgroups, NK cells, and immunoglobulins decreased after induction chemotherapy in the control group as compared with pretreatment levels, indicating that chemotherapy has an inhibitory action on immunologic function; however, among patients in the treatment group, the T-cell subgroups, NK cells, and immunoglobulin levels were significantly higher than those of the control group, suggesting that CKI protects the immune functions of patients who undergo chemotherapy, which is similar to previous research results. In addition, after induction chemotherapy, the treatment group QoL improvement rate was higher than that in the control group. Therefore, we conclude that CKI aids antitumor therapy, resists the toxicity of chemotherapy drugs, and improves the QoL by increasing the immune system function in patients with AL who receive chemotherapy. Also, our results showed that the treatment group CR and ORR were higher than those in the control after 1-2 cycles of induction chemotherapy, whereas the long-term effect regarding treatment response and survival needs further investigation. During induction chemotherapy, no any special adverse events were observed and no treatment was stopped because of adverse events of CKI in the treatment group, and the incidence of grades 3 and 4 chemotherapy-related toxicity was quite low in the treatment group than in the control, which means that CKI is safe for patients with AL and its primary active components oxymatrine and matrine have no direct interactions with used chemotherapies prescription drugs.

Traditional Chinese Medicine (TCM) is mostly prescribed in combination to obtain synergistic effects and reduce possible adverse reactions [28, 29]. Hence, the compatibility of Chinese medicinal herbs is an important theory in the combination of TCM. According to the record of Chinese herbal medicine toxicology database, Sophorae flavescentis root and seeds are poisonous; the symptom of poisoning is given priority with the nervous system, which performed for salivation, breathing and pulse acceleration, gait instability, serious eclampsia, or death from respiratory depression [30]. So reducing the toxicity of Sophorae flavescentis radix is helpful to improve the safety of clinical medication. Glycyrrhizae radix is prescribed in many Chinese traditional formulas for its medical potential effects in anti-inflammation [31], immunoregulation [32], and antiallergy nature [33]. Long-term clinical practice has also confirmed that Glycyrrhizae radix has certain detoxification effect for a variety of poisoning from drugs, animals, or the body's metabolic products [34]. In addition, after compatibility of Sophorae flavescent radix and Glycyrrhizae radix, the mortality of mice was reduced, and there were changes in the content of four kinds of indicator elements, brain tissue, and liver tissue pathology [35, 36]. Future studies may combine them together to determine the interaction of Sophorae flavescentis radix and Glycyrrhizae radix.

We acknowledge that there are some limitations to the current study. First, patients of all ages were enrolled, which resulted in a heterogeneous distribution of adjuvant treatment procedures. Additionally, the duration of follow-up was too short for CKI. We adjusted the treatment programs for patients who did not achieve CR within 2 cycles, and patients with CR received consolidation therapy or autologous or allogeneic stem cell transplantation and were no longer evaluated in the study. We may state that induction chemotherapy is only one important part of leukemia treatment, and follow-up treatment is directly related to survival, for which there may also be many uncertain factors closely based on mutations related to the patients themselves.

## 5. Conclusions

In summary, CKI combined with proven induction chemotherapy regimens for AL appears to have better short-term efficacy and lower toxicity, which may depend on increasing the patient's immunologic function and improving the QoL. In Chinese clinics, we commonly administer CKI to synergize the therapeutic effects of chemotherapy or radiotherapy. Whether CKI can prolong the survival of patients with AL requires further investigation.

## Supplementary Material

A representative fingerprint of compound Kushen injection (CKI) shows 7 common peaks: peak 1, guava base; peak 2, macrozamin; peak 3, oxymatrine; peak 4, oxysophocarpine; peak 5, N-methylcytisine; peak 6, matrine, and peak 7, sophocarpine.

## Figures and Tables

**Table 1:** Comparison of main status between treatment and control groups (*n*).

Variable	Treatment group (*n* = 167)	Control group (*n* = 165)	*χ* ^2^	*p*
Age (years)				
≤60	78	74	0.115	0.734
>60	89	91		

Sex				
Men	87	88	0.015	0.821
Women	80	77		

AML				
M1	3	4	0.306	0.989
M2	11	10		
M4	39	40		
M5	29	28		
M6	6	5		

ALL				
L1	44	44	0.013	0.993
L2	26	25		
L3	9	9		

KPS				
≥90	17	21	1.299	0.522
70–90	116	105		
≤70	34	39		

Notes: AML: acute myeloid leukemia, ALL: acute lymphocytic leukemia, and KPS: Karnofsky Performance Status.

**Table 2:** Comparison of immune function indices and cytokines in the treatment and control groups before and after treatment (*x* ± *s*).

Variable	Treatment group (*n* = 167)		Control group (*n* = 165)	
	Before treatment	After treatment	Before treatment	After treatment
CD3+	56.43 ± 4.22	55.45 ± 4.22^*∗*a^	55.38 ± 5.33	34.29 ± 4.93^b^
CD4+	31.55 ± 5.33	35.35 ± 4.24^*∗*a^	33.19 ± 5.39	17.13 ± 5.81^b^
CD8+	13.32 ± 2.33	13.12 ± 2.23^*∗*^	14.31 ± 3.02	15.23 ± 3.03^b^
CD4+/CD8+	1.69 ± 0.33	1.73 ± 0.22^*∗*^	1.68 ± 0.42	1.08 ± 0.33^b^
NK cells	15.44 ± 3.35	15.41 ± 4.02^*∗*^	15.12 ± 3.72	10.12 ± 3.11^b^
IgG	12.5 ± 1.3	12.3 ± 1.2^*∗*^	12.4 ± 1.3	7.2 ± 1.5^b^
IgA	2.4 ± 0.3	2.3 ± 0.3^*∗*a^	2.4 ± 0.3	1.2 ± 0.5^b^
IgM	1.71 ± 0.30	1.58 ± 0.4^*∗*a^	1.81 ± 0.47	0.45 ± 0.53^b^
IL-4	20.0 ± 2.4	22.2 ± 2.3^*∗*a^	22.5 ± 3.2	11.8 ± 2.2^b^
IL-10	17.10 ± 2.2	19.4 ± 4.2^*∗*a^	18.90 ± 2.1	9.80 ± 3.2^b^

Notes: compared with the control group after treatment, ^*∗*^ *p* < 0.05; compared with the treatment group before treatment, ^a^ *p* < 0.05; compared with the control group before treatment, ^b^ *p* < 0.05.

**Table 3:** Response to induction chemotherapy in the treatment and control groups.

Variable	AML (%)		ALL (%)	
	Treatment group (*n* = 88)	Control group (*n* = 87)	Treatment group (*n* = 79)	Control group (*n* = 78)
CR (%)	72 (81.8)	58 (66.7)	64 (81)	51 (65.4)
CRi (%)	9 (10.2)	6 (6.9)	7 (8.9)	3 (3.8)
ORR (%)	81 (92)^a^	64 (73.6)	71 (89.9)^a^	54 (69.2)

Notes: AML: acute myeloid leukemia, ALL: acute lymphocytic leukemia, CR: complete remissions, CRi: CR with incomplete blood count recovery, and ORR: overall response rate. Compared with the control group, ^a^ *p* < 0.05.

**Table 4:** KPS index changes of two groups before and after treatment.

Variable	AML (%)		ALL (%)	
	Treatment group (*n* = 88)	Control group (*n* = 87)	Treatment group (*n* = 79)	Control group (*n* = 78)
Improvement (%)	49 (55.7)^a^	25 (28.7)	47 (59.5)^a^	19 (24.4)
Stabilization (%)	31 (35.2)	37 (42.5)	23 (29.1)	29 (37.2)
Deterioration (%)	8 (9.1)^a^	25 (28.7)	9 (11.4)^a^	30 (38.5)

Notes: AML: acute myeloid leukemia, ALL: acute lymphocytic leukemia. Compared with the control group, ^a^ *p* < 0.05.

**Table 5:** Grade 3/4 drug toxicity and adverse events occurring during the induction period in the treatment and control groups.

Variable	AML (%)		ALL (%)	
	Treatment group (*n* = 88)	Control group (*n* = 87)	Treatment group (*n* = 79)	Control group (*n* = 78)
Hematologic toxicity	6 (6.8)^a^	21 (24.1)	3 (3.8)^a^	17 (21.8)
Gastrointestinal reaction	2 (2.3)^a^	10 (11.5)	1 (1.3)^a^	8 (10.3)
Pneumonia	1 (1.1)^a^	9 (10.3)	1 (1.3)	6 (7.7)
Cardiotoxicity	0 (0)	4 (4.6)	1 (1.3)	5 (6.4)
Hepatotoxicity	3 (3.4)^a^	11 (12.6)	2 (2.5)^a^	9 (11.5)
Neurological dysfunction	1 (1.1)^a^	7 (8)	1 (1.3)^a^	8 (10.3)
Renal dysfunction	3 (3.4)^a^	16 (18.4)	1 (1.3)^a^	11 (14.1)
Skin reactions	1 (1.1)^a^	7 (8)	0 (0)^a^	5 (6.4)

Notes: AML: acute myeloid leukemia, ALL: acute lymphocytic leukemia. Compared with the control group, ^a^ *p* < 0.05.
