# Supplementary material for: Efficacy of Compound Kushen Injection in Combination with Induction Chemotherapy for Treating Adult Patients Newly Diagnosed with Acute Leukemia
Source: Evid Based Complement Alternat Med. 2016 Sep 21;2016:3121402. doi: 10.1155/2016/3121402 (PMC5050378; doi:10.1155/2016/3121402)
Supplement: Supplementary file 1 — A representative fingerprint of compound Kushen injection (CKI) shows 7 common peaks: peak 1, guava base; peak 2, macrozamin; peak 3, oxymatrine; peak 4, oxysophocarpine; peak 5, N-methylcytisine; peak 6, matrine, and peak 7, sophocarpine. [file 3121402.f1.pdf]

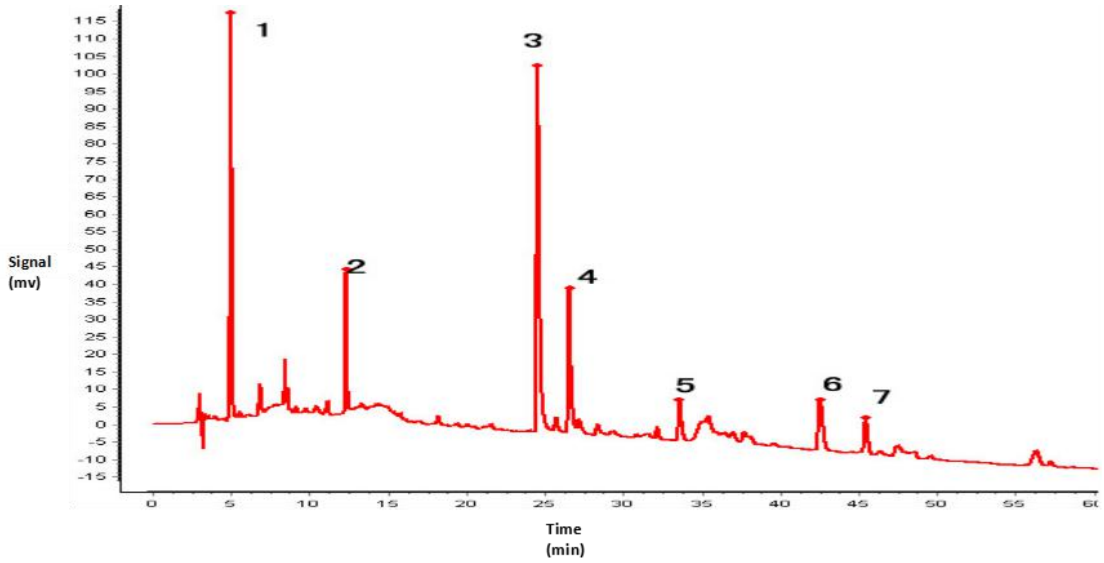

Additional file 1: A representative fingerprint of CKI.

A representative fingerprint of CKI showing 7 common peaks: peak 1, guava base; peak 2, macrozamia; peak 3, oxymatrine; peak 4, oxysophocarpine; peak 5, caulophylline; peak 6, matrine, and peak 7, sophocarpine.
